# SOCIAL NETWORKS AND COGNITIVE FUNCTION: POLICY IMPLICATIONS FROM RECENT RESEARCH

**DOI:** 10.1093/geroni/igac059.1583

**Published:** 2022-12-20

**Authors:** William McConnell, Katherine Haggar, Mohit Manchella, Adam Roth, Brea Perry

**Affiliations:** Florida Atlantic University, Boca Raton, Florida, United States; Florida Atlantic University, Boca Raton, Florida, United States; University of Southern Indiana, Carmel, Indiana, United States; Indiana University, Bloomington, Indiana, United States; Indiana University, Bloomington, Indiana, United States

## Abstract

This presentation will describe a series of research projects using Social Network Analysis (SNA) to measure the personal social networks of community-dwelling older adults at-risk for or currently experiencing dementia. The first part of the presentation will provide a brief overview of SNA methods, including advantages of SNA compared to more traditional social activity scales. The second part of the presentation will present evidence from multiple studies to identify distinct pathways linking upstream social network characteristics to downstream pathophysiological processes in the aging brain. In particular, we will distinguish between cognitive stimulation experienced in expansive social networks and neuroendocrine benefits derived from cohesive social networks. The third part of the presentation will present implications for psychosocial interventions to reduce health disparities in dementia care and prevention. We recommend leveraging the multidimensional functionality of social networks across the lifecourse to influence multiple cognitive health pathways simultaneously.

